# Chronic Pain in the Elderly: Mechanisms and Perspectives

**DOI:** 10.3389/fnhum.2022.736688

**Published:** 2022-03-03

**Authors:** Ana P. A. Dagnino, Maria M. Campos

**Affiliations:** ^1^Programa de Pós-graduação em Medicina e Ciências da Saúde, Escola de Medicina, Pontifícia Universidade Católica do Rio Grande do Sul, Porto Alegre, Brazil; ^2^Centro de Pesquisa em Toxicologia e Farmacologia, Escola de Ciências da Saúde e da Vida, Pontifícia Universidade Católica do Rio Grande do Sul, Porto Alegre, Brazil; ^3^Programa de Pós-graduação em Odontologia, Escola de Ciências da Saúde e da Vida, Pontifícia Universidade Católica do Rio Grande do Sul, Porto Alegre, Brazil

**Keywords:** chronic pain, elderly, mechanisms, perception, management, life quality

## Abstract

Chronic pain affects a large part of the population causing functional disability, being often associated with coexisting psychological disorders, such as depression and anxiety, besides cognitive deficits, and sleep disturbance. The world elderly population has been growing over the last decades and the negative consequences of chronic pain for these individuals represent a current clinical challenge. The main painful complaints in the elderly are related to neurodegenerative and musculoskeletal conditions, peripheral vascular diseases, arthritis, and osteoarthritis, contributing toward poorly life quality, social isolation, impaired physical activity, and dependence to carry out daily activities. Organ dysfunction and other existing diseases can significantly affect the perception and responses to chronic pain in this group. It has been proposed that elderly people have an altered pain experience, with changes in pain processing mechanisms, which might be associated with the degeneration of circuits that modulate the descending inhibitory pathways of pain. Aging has also been linked to an increase in the pain threshold, a decline of painful sensations, and a decrease in pain tolerance. Still, elderly patients with chronic pain show an increased risk for dementia and cognitive impairment. The present review article is aimed to provide the state-of-art of pre-clinical and clinical research about chronic pain in elderly, emphasizing the altered mechanisms, comorbidities, challenges, and potential therapeutic alternatives.

## General Concepts and Pain Mechanisms

Currently, pain is defined as “an unpleasant sensory and emotional experience associated with, or resembling that associated with, actual or potential tissue damage” (Raja et al., 2020). Pain is an essential defense mechanism in mammals, alongside harmful environments or behaviors, favoring their survival. Pain can be divided into two main types: acute and chronic pain. Acute pain aims to prevent our organism from being exposed again to injuries, while chronic pain manifests itself in a pathological, inadequate manner, causing suffering to the affected individuals (Kuner and Flor, 2016). As discussed along this review article, chronic pain can represent a great limitation for elderly people.

Pain begins with the stimulation of nociceptors, which are skin fibers able to detect peripheral stimuli through their molecular sensors arranged in the nerve terminals, carrying the signs to the dorsal horn of the spinal cord via the dorsal root ganglia (DRG). The nociceptors are located on three classes of primary nerve fibers involved in pain transmission, namely Aβ, Aδ, and C afferents. Nociceptors are specialized in distinguishing stimuli from different sources, such as mechanical, thermal, or chemical insults, in addition to detect immune mediators (bradykinin, cytokines, and histamine), ATP, microorganisms, and their toxins. Subsequently, the stimulus is transduced into an action potential and processed in the brain, becoming perceived as a pain sensation (Basbaum et al., 2009). Interestingly, neuroimmune interactions occur at both the peripheral and central levels, modulating pain during inflammation. Macrophages, mast cells, neutrophils, and T cells act in the peripheral nerve sensitization through the release of cytokines, growth factors, chemokines, and lipid mediators. The central mechanisms of pain can be modulated by neuroimmune interactions in the spinal cord. At this stage, pain neurotransmission involving the production of ATP, cytokines, and glutamate is likely influenced by the crosstalk between T cells, microglia, and astrocytes that act on pre and post-synaptic neurons (Baral et al., 2019).

It is well known that some active lipids are positively regulated in acute and chronic pain, including prostaglandin E2 (PGE_2_), prostacyclin (PGI_2_), dihydroxyeicosatrienoic acids (DHETs), lysophosphatidic acid (LPA), platelet activating factor (PAF), and sphingosine 1 phosphate (S1P). Recent studies demonstrated that LPA is involved in the onset and maintenance of chronic pain in animal models of pain. Initially, the nerve injury causes the activation of phospholipase A2 (PLA2), resulting in the production and the extracellular release of lysophosphatidylcholine (LPC), and the conversion to LPA. Microglia is activated by LPA and produces interleukin 1β (IL-1β) and brain-derived nerve factor (BDNF). IL-1β activates spinal dorsal horn neurons and stimulates PLA2, leading to a feed-forward LPA production. BDNF production triggers the conversion of GABA_*A*_ receptor function from an inhibitory outline to an excitatory state. Collectively, these mechanisms are involved in allodynia and hyperalgesia in neuropathic pain (Ueda, 2021). Activated microglia releases tumor necrosis factor (TNF), which in turn activates nearby astrocytes. The astrocytes induce the sensitization of primary afferents and the excitation of spinal dorsal horn nociceptors, through the release of IL-1β and interleukin-18 (IL-18), as well as Cx43-mediated release of ATP, glutamate, and the chemokines C-X-C motif ligand 1 (CXCL1) and C-C motif chemokine ligand 2 (CCL2) (Ji et al., 2019; Donnelly et al., 2020). After nerve injury or noxious stimulation, astrocytes become reactive astrocytes A1 and secrete neurotoxins that induce the death of neurons and oligodendrocytes (Li et al., 2019). The chemokines and their receptors exert a pivotal role in chronic pain conditions, via enhanced neuroinflammation in the peripheral nerves, DRG, spinal cord, and brain. Several chemokines mediate an interaction between neurons and non-neuronal cells, or neurons with inter-neurons, participating in the pathogenesis of neuropathic, inflammatory, cancer-related, visceral, and dysfunctional pain (Jiang et al., 2020). Oligodendrocytes can modulate pain through interleukin-33 (IL-33) expression, under peripheral neuropathy. In turn, IL-33 activates microglia, with the ensuing release of proinflammatory cytokines, such as TNF and IL-1β, with hyperalgesia induction (Malta et al., 2019).

Recently, the molecular mechanisms for microbial-driven pain have been described. Products derived from gram-positive and negative bacteria, fungal or skin pathogens are detected by peripheral nociceptors by different sensors (some of them well recognized, such as TLR4 and 5, TRPA1, TRPV1, AT2R, FPR1, and toxin pore assembly). Herpes viruses, when invading neurons, are also capable of modulating and causing pain. The pathogen-driven stimulus is recognized by nociceptors and immune cells that then respond directly to pain signal transduction (Baral et al., 2019). Gut microbiota-derived mediators can regulate peripheral sensitization of pain, either positively or negatively. For instance, bile acids (BA) can inhibit pain, inducing opioid release from immune cells (macrophages) and decreasing the DRG neuron excitability. Alternatively, BA can activate the farnesoid X receptors (FXR), prompting mast cells to release the nerve growth factor (NGF), and augment the pain intensity (Guo et al., 2019).

During the pain processing, different regions of the central nervous system (CNS) are activated. Neural activation mainly occurs in the primary somatosensory cortex (S1), secondary somatosensory cortex (S2), anterior cingulate cortex (ACC), insula, prefrontal cortex (PFC), thalamus, and cerebellum (Apkarian et al., 2005). Nociceptive stimulus reaches to the supraspinal areas, mostly specifically the amygdala and nucleus accumbens, and the periaqueductal gray region (PAG), through spinoparabrachial and spinoreticular pathways, respectively (Basbaum and Fields, 1984; Bernard et al., 1996; Becerra et al., 2001; Dunckley et al., 2005; Baliki et al., 2010). The ACC and the insula areas (the limbic system) are involved in the affective component of pain, while the S1 and S2 are sensory regions, which are pivotal for determining the pain duration and location. Emotional and cognitive states directly influence the chronic pain neural mechanisms and an increased pain negatively modulate the individual’s emotional and cognitive states. These two factors act on afferent nociceptive signals to the brain and descending inhibitory pathways of pain. Interestingly, emotional, and cognitive states modulate pain perception differently. The altered emotional state (mood) modulates the pain unpleasant sensation by activating circuits in the ACC, PFC, and PAG. Alternatively, modified cognitive status (attention) regulates the pain perception intensity through the activation of pathways in the superior parietal lobe (SPL) to the S1 and the insula (Bushnell et al., 2013).

The study of changes in mechanisms of pain transmission in aging represents a relevant field of research. In the next sections, we present some of the recent advances concerning the peculiar features of pain perception in the elderly population, aiming to highlight the epidemiology and some especial conditions in which pain aggravates other diseases in older individuals.

## Chronic Pain Burden in the Elderly

Consistent with the current concepts, chronic pain is defined as “pain that lasts or recurs for longer than 3 months,” being classified as primary (such as fibromyalgia)—when the pain is the disease itself, or secondary—when it is related to another previous illness (e.g., cancer-related pain) (Treede et al., 2019). Mostly, the main causes of chronic pain in the elderly are secondary to another previous disorder, encompassing cancer, neuropathic pain, musculoskeletal changes, chronic post-traumatic or postsurgical pain, chronic visceral pain, chronic headache, and orofacial pain, as reviewed by Zis et al. (2017). According to a systematic review published in 2019, the prevalence of low back pain among the elderly population ranged from 21 to 75% and this pain condition led to functional disability in 60% of the studies (de Souza et al., 2019). The chronic back pain occurred in 20.7% of adults or elderly from Southern Brazil and it was associated with poorer health perception, poorer quality of life, and depressive symptoms (Saes-Silva et al., 2021). An overview of persistent pain in older adults showed the prevalence of osteoarthritic back pain, especially in the low back or neck (around 65%), musculoskeletal pain (around 40%), peripheral neuropathic pain (typically due to diabetes or postherpetic neuralgia, 35%), and chronic joint pain (15–25%) as reviewed by Molton and Terrill (2014).

Regardless of the type, there has been an increase in the numbers of middle-aged and older adults with chronic pain in the last decades. Striking, the estimated prevalence of chronic pain is higher as 25–50% in community-dwelling elders, reaching up to 80% in institutionalized individuals (Cravello et al., 2019). One factor that had been associated with this rise is the obesity epidemics, with the body mass index (BMI) being considered a risk factor for increased mild to severe pain trends in women and men (Stokes et al., 2020). Another study proposed that both, an older age, and the presence of at least one comorbidity, were predicting factors for chronic musculoskeletal pain in elders (Kirubakaran and Dongre, 2019). An interesting longitudinal cohort analysis, carried out with oldest-old individuals (>75-year-old), suggested that peripheral arterial disease, low back pain, high BMI, and female sex are associated with a higher risk of pain experience at old ages, whereas smoking cessation might account for a reduction of pain syndromes in seniors (Mallon et al., 2019). As reviewed by Domenichiello and Ramsden (2019), aging can be considered as a risk factor for chronic pain, being a primary cause of disability or a consequence of other diseases commonly seen in geriatric patients. Persistent pain in the elderly has huge impacts on health care system costs due to the complexity of treatment, and exacerbation of psychological conditions, comprising anxiety, depression, insomnia, and poor life quality levels. Remarkably, the authors point out that long-lasting pain might represent a risk factor for mortality in elders, worsening common problems, such as cognitive deficits and insufficient social interaction (Domenichiello and Ramsden, 2019). Additional studies of pain in elderly will help to pave the way regarding the gaps in pain prevalence and incidence in this population. Therefore, innovative methods for suitably assessing pain rates and manifestations in this population remain an issue to be developed in a near future. This is relevant for improving pain diagnosis and for guiding interdisciplinary teams in the management of those individuals, aiming to reach global health benefits. In the next sections, recent evidence regarding chronic pain in the elderly will be discussed, mainly focusing on the mechanisms linking aging and pain, besides therapeutic options for the elderly.

## Pain Transmission and Experience in the Geriatric Population

This section was intended to discuss the recent advances relating to the odd aspects of chronic pain states in the elderly. An interesting bibliometric study, based on articles published between 2000 and 2019 (Zhao et al., 2021), revealed that most papers concerning pain in the elderly are related to original research on pain characterization, with limited evidence regarding the biological factors that are particularly relevant for pain processing in aging. This is a clear indicative that the study of pain transmission in old people is a theme of current interest and an actual challenge, justifying the discussion of this topic in the present review article. In this regard, most of the studies evaluate pain processing and perception in the elderly, by comparing the outcomes in relation to younger controls, either in pre-clinical or clinical studies. That said, there is no doubt about the intricacy of characterizing pain in the old population. In fact, age-related changes regarding the sensorial and affective components of pain remain controversial.

Elderly people tolerate acute pain better when compared to persistent pain. This might be correlated with differences in duration and origin/cause of chronic and acute pain. Sensory mechanisms (defective neuroplasticity or impaired nociceptive pathways), behavioral components (such as superior pain acceptance and self-efficacy, and less catastrophizing levels), as well hormonal (estrogen), and social factors (social support) can influence pain perception in the elderly. Furthermore, the mechanisms underpinning these altered mechanisms needs further investigation to be solved.

**FIGURE 1:**
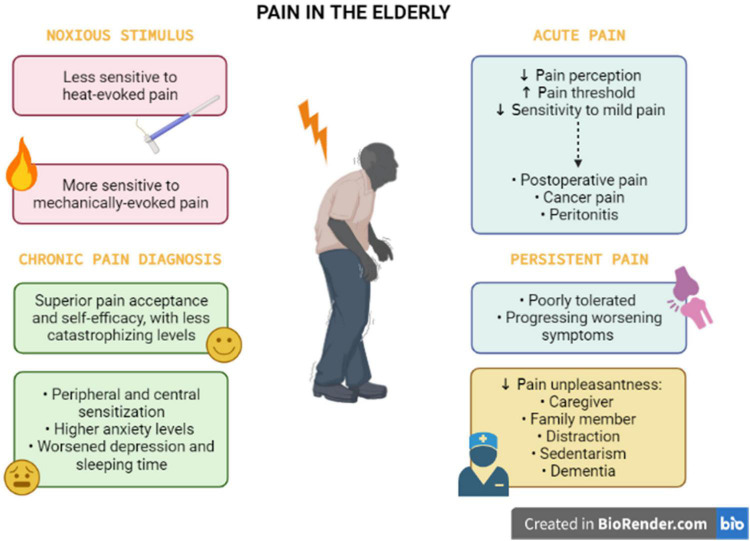
Pain perception and experience in the elderly. The pain perception in the elderly is complex, with biopsychosocial factors associated. The older individuals present opposite sensitivity for heat and mechanically evoked pain. This population apparently tolerates acute pain (postoperative and cancer pain, peritonitis), when compared with persistent pain states. For chronic pain diagnosis in the elderly, health professionals should consider that older adults demonstrate particularities such as superior pain acceptance and self-efficacy, with less catastrophizing levels. However, the elderly has altered peripheral and central sensitization combined with higher levels of anxiety, depression, and insomnia. Social support from a family member or a caregiver, distraction, sedentary life, and dementia can influence the pain experience of older people, leading to changes in pain unpleasantness.

The interplay between pain perception and aging is still unknown. Firstly, it is important to understand that the experience of pain in older adults is modeled by changes in biological, psychological, and social factors that occurred during aging events. Aging is related to decreased pain perception in several pain conditions affecting older adults, such as postoperative pain, cancer pain, and peritonitis. Pain has a warning function, to avoid the tissue damage against a noxious stimulus. This function might be compromised in older persons that have an augmented pain threshold and a reduced sensitivity to mild pain, leading to increased injury and undiagnosed disease (Gibson and Helme, 2001). In contrast to acute pain, persistent pain is poorly tolerated by elderly people. In older persons, the impact of persistent symptoms of hip or knee pain is substantial, frequently progressing in terms of worsening symptoms and accrual of other symptomatic hip or knee joints (Dawson et al., 2005). As reviewed by El Tumi et al. (2017), old adults may be less sensitive to heat-evoked pain and more sensitive to mechanically evoked pain, when compared with young adults. A systematic review and meta-analysis reported that the average pain threshold was significantly increased in older people (>60 years), when compared with younger individuals, in response to thermal, electrical, and pressure stimulation, besides rectal and esophageal distension. In relation to differences in pain tolerance thresholds, the average values did not significantly differ between younger and older groups, despite the difference trended to be significant (Lautenbacher et al., 2017; see Figure 1). Based on these pieces evidence, it is clear that pain perception is highly impacted by aging process itself, but it is also dependent on the presence of other common diseases in the old population.

The progressive degeneration of intervertebral discs along with aging has been linked to chronic low back pain. It was demonstrated that microRNAs and altered autophagy processes are implicated in intervertebral disc deterioration in seniors. Allied with increased levels of inflammatory mediators, such as cytokines and metalloproteinases, these mechanisms might account for aggravating lumbar painful symptoms in elders (Lan et al., 2021). A clinical study demonstrated that young individuals with chronic low back pain exhibits a reduction of musculoskeletal proprioception, an effect that was seen in older adults regardless of the diagnosis of chronic lumbar pain. This is indicative that aging is related to an impairment of peripheral and central mechanisms underlying proprioceptive transmission (Pinto et al., 2020), what might account for development of chronic low back pain in the elderly. Alternatively, chronic pain has been considered a risk factor for fall in elders, likely by affecting the reflex responses. A cross-sectional study enrolling more than 300 institutionalized elders (MOBILIZE Boston Study II) demonstrated that severity of musculoskeletal pain was associated with an increased time for simple foot reaction (Cai Y. et al., 2021). Another study evaluating the same group of individuals revealed that pain negatively influenced all the gait measures, mainly the gait speed, probably via alterations of cognitive pathways (Ogawa et al., 2020). Data points out complex neuromotor mechanisms linking chronic pain and an increased risk of falls in older individuals. Moreover, pain control might also prevent common vulnerabilities in the elderly, helping in prevention of falls and the related consequences, besides improving the overall mobility of the old individuals with chronic pain. Thus, it is tempting to suggest that chronic pain can aggravate the aging-related frailty complications in elderly.

### Chronic Pain and Aging

A recent pre-clinical study conducted by Geltmeier and Fuchs (2021) suggested that older rats displayed an impaired avoidance behavior in response to chronic pain in the arthritis model elicited by complete Freund’s adjuvant (CFA), despite an increased pain threshold for mechanical and thermal stimulation in relation to younger rats. This evidence allows suggesting that elders might have poorer outcomes concerning the emotional processing of pain, regardless of the reduction in pain sensitivity. In contrast, a clinical study revealed that older adults aging from 65 to 75 years, presenting with chronic pain diagnosis, had a superior pain acceptance and self-efficacy, with less catastrophizing levels, in comparison with middle-aged and young adults (Murray et al., 2021). Thus, older individuals apparently perform better with pain experiences, with inferior levels of pain-related depression and disability when contrasted with younger individuals. Based on this data, it is feasible to propose that additional studies are still required to understand age-related changes of sensorial mechanisms and behavioral components of chronic pain. Nonetheless, the older population exhibits greater differences in pain perception in relation to youngers and this must be considered for pain management strategies (see Figure 1).

One of the most common conditions leading to chronic pain and disability in the elderly is osteoarthritis. This is likely related to the obesity burden, combined with the senescence of connective tissues during aging, which might result in painful alterations at activity or rest (Satake et al., 2021). Frailty has also been associated with osteoarthritis in the elderly, likely via the activation of inflammatory pathways, being osteoarthritis-related pain an aggravating factor for frailty severity itself (O’Brien and McDougall, 2019). Unfortunately, the current treatments for pain relief are associated with several side effects, which is a shortcoming for long-term therapy. Thus, there is an urgent need for newer innovative drugs able to control inflammatory changes, besides painful sensation in aged individuals with osteoarthritis. Recent concepts propose individualized treatments based on the phenotyping of the disease. However, this idea is quite incipient, and it requires further advancements to reach the clinical set (Cai X. et al., 2021). Some relevant biomarkers of osteoarthritis-related pain in older individuals have also been identified in the last years, what might account for future advances in pain control strategies. For instance, it has been demonstrated that plasma levels of BDNF positively correlate with heat pain threshold and numerical pain scale in old individuals with osteoarthritis diagnosis (Sorkpor et al., 2021). BDNF might well represent a target to be explored attempting to identify new effective alternatives for pain management in elderly.

A cross-sectional clinical study enrolling a hundred of > 60-year-old individuals, with knee osteoarthritis and dysfunctional activity of endogenous pain-inhibitory system, indicated that structural articular damage was associated with both peripheral and central sensitization, and higher anxiety levels. It has also been shown that knee joint pain worsened depression and sleeping time, impairing the health-related life quality levels. Altogether, joint deterioration, depressive state and higher pain perception led to weakened physical activity levels (Tavares et al., 2020). Hence, chronic pain in the elderly is probably associated with a deficiency of pain-induced pain relief mechanisms, with a direct relationship with physical disability, sleep disturbances, depression, and anxiety, while compromising the life quality of the affected subjects. This clearly indicates that the intricate pain perception mechanisms in the elderly involve a myriad of factors that influence each other, including changes of affective dimensions, increasing the complexity of treating those patients.

### Factors That Aggravate Pain in Elderly

Changes in central mechanisms of pain transmission have been characterized by functional magnetic resonance imaging (fMRI) in the model of osteoarthritis induced by monosodium iodoacetate in rats. The authors verified that older females displayed an enhanced activation of PAG connections in the late phase of the model, with an involvement of ACC (Da Silva et al., 2021). This might explain previous data from the same group showing that older female rats present an exaggerated and long-lasting hyperalgesia response after induction of osteoarthritis by monosodium iodoacetate, when compared with young male mates (Ro et al., 2020). The authors have also demonstrated that old rats, mainly females, display a reduction of endogenous inhibitory pathways of pain, likely via an involvement of the limbic system, by evaluating the thermal withdrawal responses after capsaicin injection (Da Silva et al., 2020). Collectively, these results imply that age-related changes of PAG networks are implicated in defective pain control circuits in elderly. An interesting set of evidence demonstrated that dorsal horn neurons obtained from old mice exhibited faster action potential discharges, with a reduction of excitatory inputs allied to an increased GABAergic inhibitory signaling, in comparison with the younger ones, according to evaluation by patch clamp electrophysiology (Mayhew et al., 2019). The authors suggested that aged spinal neurons might be more susceptible to injuries, helping to explain the altered pain transmission in the elderly. Pharmacological and non-pharmacological strategies focused on the modulation of those central pathways might be useful for the management of chronic pain in elders in a near future. Further brain imaging studies are of high relevance to gain new insights concerning the particular central pathways implicated in pain transmission in older individuals.

### Factors That Alleviate Pain in Olders

Social support from a family member or a caregiver can distinctly influence the pain experience in the elderly. The presence of a caregiver decreases the pain unpleasantness, when compared with individuals living alone. However, the family condition can induce significantly more intense non-verbal pain expressions than the caregiver condition. Strikingly, the same study demonstrated that older males had an increased pain tolerance when compared with older females (Gallant and Hadjistavropoulos, 2017). The sex differences relating to pain perception in older persons have also demonstrated by Romano et al. (2019). The authors reported that females have significantly less unpleasantness with moderate pain perception (contact to heat), when compared with males. Other important aspect is the residence local of older individuals. A group of researchers demonstrated that older adults living in nursing homes perceived more pain in relation to the home care group (Xu et al., 2018). Another evidence regarding the primordial role of social support in alleviating pain is the animal-assisted therapy. This intervention was effective to decrease pain perception and pain-related insomnia in poly-medicated geriatric patients with chronic joint pain (Rodrigo-Claverol et al., 2019). A recent study showed pain relief during distraction in older and younger participants. However, the older group presented higher ratings of unpleasantness to painful stimulation and attenuated neural responses, regardless of the distraction condition (Gonzalez-Roldan et al., 2020). Of note, an association concerning sedentarism and chronic pain modulatory mechanisms in the elderly has been proposed. Accordingly, a cross-sectional study showed a positive correlation between sedentary behavior and pain pressure thresholds in middle-aged and older adults with musculoskeletal pain diagnosis (Mani et al., 2019). As proposed by the authors, sedentarism might represent a protective attitude against pain sensation, despite new investigations are required in this regard (see Figure 1).

On other hand, clinical trials demonstrated that pain crisis is reduced in pain types in which estrogens display a relevant role. For instance, increasing age has been negatively associated with abdominal pain severity in a sample of women aging from 35 to 55 years. The authors attributed this effect to decreased estrogen levels as women move from late reproductive to post menopause stages (Callan et al., 2019). In addition, menopause also contributes to a reduction of headache and cervical/lumbar pain symptoms (Meriggiola et al., 2012). In agreement, it has been well established in the literature that the different sex hormone status contributes to higher and lower migraine prevalence, when comparing women in reproductive age and before puberty and postmenopausal stages, respectively (Chai et al., 2014).

## Pain Associated With Specific Conditions in Old Ages

Long-lasting unresolving disorders (such as dementia, inflammatory pain, or viral infections) produce ongoing nociceptive or neuropathic stimuli and might be entirely responsible for chronic pain in the elderly. The psychological factors can increase persistent pain; besides, chronic pain can exacerbate psychological problems (e.g., depression), impairing the differentiation between the cause and the effect. Parkinson’s and Alzheimer’s diseases, depression, burning mouth syndrome, and viral infections are particularly relevant for chronic pain, and their presence might have an impact on pain and physical function in geriatric patients. Compelling data demonstrate the need for investigation into the treatment of these comorbidities in older patients with chronic pain. Some of the aspects linking pain and age-related maladies will be discussed in the next items.

### Neurodegenerative Diseases

**FIGURE 2:**
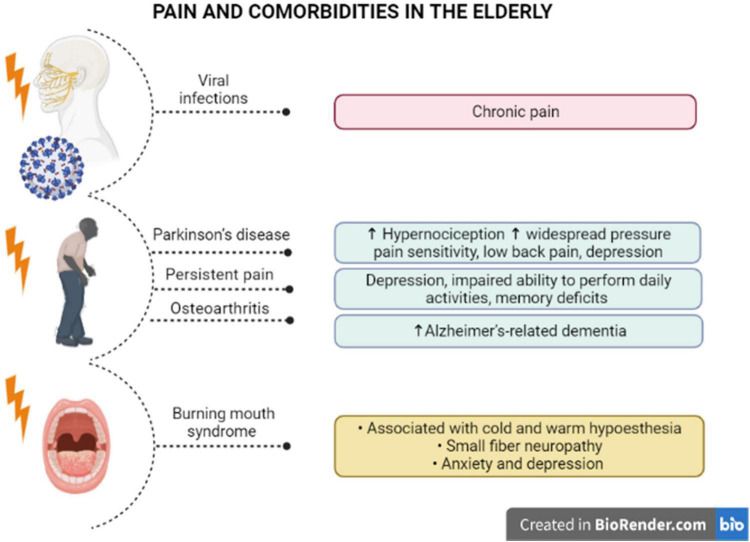
Pain and specific comorbidities in the elderly. There is evidence of a correlation between pain syndromes and comorbidities in older individuals. Viral infections in elder patients with comorbidities has been associated with chronic pain. Parkinson’s disease induces higher hypernociception and widespread pressure pain, low back pain, and depression, and pain likely precedes the motor symptoms. Also, persistent pain has been related to the appearance of depression and memory deficits in older people. It has been proposed that elders with osteoarthritis are at higher risk to develop Alzheimer’s dementia. Older women with burning mouth syndrome diagnosis demonstrated cold and warm hypoesthesia, small fiber neuropathy, with higher levels of anxiety and depression, in a condition that is typically misdiagnosed in seniors.

The painful symptoms in elders must be carefully investigated, even as an indicative of neurodegenerative disorders. Some pieces of evidence have demonstrated that hypernociception precedes the development of motor symptoms in Parkinson’s disease. A pre-clinical study showed that induction of Parkinsonism by reserpine in middle-aged rats triggered mechanical and chemical hypersensitivity, besides amine depletion and c-FOS activation, after induction of chemical nociception (Cintra et al., 2021). Noteworthy, elders with Parkinson’s disease manifested higher widespread pressure pain sensitivity, after bilateral pressure application to the cervical spine, the second metacarpal, or the tibialis anterior, with lower pain thresholds for females than males (Ferreira-Sanchez et al., 2020). Moreover, a cross-sectional study enrolling 115 individuals with Parkinson’s disease showed that most of them (>80%) had painful symptoms, with almost 60% of the cases corresponding to low back pain (Silveira Barezani et al., 2020). Some conclusions raised in this study merit to be emphasized: (i) about 40% of low back pain carriers had painful symptoms before the Parkinson’s diagnosis, reinforcing the idea that pain might precede neurodegeneration in Parkinson’s disease; (ii) the disease severity has been correlated with higher pain intensity, depression symptoms, disability and poor life quality, as an indication that pain can be an aggravating factor for Parkinson’s disease-related limitations, including psychological disorders. This hint was rather supported by evidence showing a significant correlation of pain intensity, pain-related disability, as well as pain interference, with depression symptoms in Parkinson’s disease (Rana et al., 2017; see Figure 2). Pain in patients carrying out Parkinson’s disease has also been linked with the severity of motor symptoms, in addition to sleep and mood disturbances, with a female predominance regarding pain symptoms (Defazio et al., 2017). The liaison between painful symptoms and Parkinson’s disease severity in the elderly might be dependent on both structural and anatomical changes of supraspinal networks, mainly involving the relative thinning of cortical regions and degenerated connections of nucleus accumbens and hippocampus (Polli et al., 2016).

One hypothesis that is currently gaining attention is that dopaminergic deficits are associated with anhedonia and chronic pain (Navratilova and Porreca, 2014; Schwartz et al., 2014; Porreca and Navratilova, 2017). The excitability of GABAergic indirect pathway spinal projection neurons was increased in spared nerve injury (SNI) model of neuropathic pain (Ren et al., 2016). The activity of these neurons is eliminated by the activation of postsynaptic dopamine D2 receptors (Hikida et al., 2010; Danjo et al., 2014). The link between pain and dopamine neurotransmission alterations was observed in chronic non-neuropathic back pain patients (CNBP), when compared to healthy controls. The D2/D3R availability in the ventral striatum was reduced in CNBP. This result was associated with greater positive scores and pain tolerance measures (Martikainen et al., 2015). This is also interesting because the dopaminergic neural mechanisms of cognitive flexibility are affected by aging and dopamine has been associated with cognition and executive functions changes in adults (Berry et al., 2016). Therefore, dopamine transmission and dopamine receptor-activated signaling pathways are relevant components to be considered in the association of Parkinson’s disease and chronic pain in seniors.

Chronic pain is also commonly observed in patients with dementia, mainly with Alzheimer’s disease diagnosis, although the influence of chronic pain on disease progression remains to be further investigated. In this regard, a longitudinal study evaluating more than 1,000 > 70-year-old community-dwelling individuals, throughout 21 years (Einstein Aging Study), demonstrated that high levels of pain interference in usual skills were associated with an elevated risk of developing all-cause and Alzheimer’s-related dementia, irrespective of no significant correlation between pain intensity and the latency for dementia (Ezzati et al., 2019). In institutionalized elders with dementia, there was a positive correlation between pain diagnosis and depression, what accounts for the complexity of pain evaluation and management in this specific population (Malara et al., 2016). Reinforcing this notion, Whitlock et al. (2017) demonstrated that community-dwelling older adults (Health and Retirement Study), presenting with persistent pain, had worsened depression symptoms and an impaired ability to perform daily activities, allied with accelerated memory deficits. Considering that this cohort included more than 10,000 patients that had been accompanied for 12 years, it was possible to assume that persistent pain led to an absolute increase of 2.2% in the probability of getting dementia (Whitlock et al., 2017). A retrospective cross-sectional analysis that included > 65-year-old persons showed that pain interference on daily activities has been positively correlated with dementia in Alzheimer’s disease, regardless of osteoarthritis diagnosis (Ikram et al., 2019; see Figure 2). Alternatively, a retrospective cohort study, enrolling > 65-year-old adults recruited from the American government health insurance program Medicare, showed that elders with a previous painful osteoarthritis were at higher risk of developing Alzheimer’s dementia, in comparison with osteoarthritis-free seniors, with a partial relevance for anxiety and depression in this connection (Innes and Sambamoorthi, 2020).

Whereas pain diagnosis in the absence of cognitive decline is mainly performed by self-assessment scales, elders with dementia require additional strategies of evaluation, such as observation of typical painful behaviors and in-depth investigation of pain origin. Additionally, the reports of caregivers and the implementation of strategies for pain relief can also provide clues for an appropriate pain diagnosis in elders with dementia (Cravello et al., 2019). During the last decade, many initiatives have been made to develop and validate instruments for pain evaluation in the senior population, while some of these surveys are specific for individuals with cognitive deficits (Andrade et al., 2011).

Noteworthy, efforts to better understand the mechanistic connections between chronic pain and dementia might facilitate the diagnosis and treatment of the affected elders in a near future. For instance, it has been proposed that noradrenergic dysfunction and neuroinflammation are common central mechanisms affecting brain regions related to both affective components of pain and cognitive networks in Alzheimer’s-affected patients (Cao et al., 2019). Besides, it has been demonstrated that acetylcholinesterase inhibitors, such as neostigmine and rivastigmine, that had been used for the management of dementia in patients with Alzheimer’s or Parkinson’s disease, display beneficial effects on chronic pain, considering that acetylcholine deficits are implicated in either cognitive impairment or persistent pain (Eldufani and Blaise, 2019). In a pre-clinical evaluation, the induction of osteoarthritis by monosodium iodoacetate in transgenic mice, with Alzheimer’s disease, led to a reduced mechanical hypersensitivity response, and a weaker spinal microglia activation, when compared with wild-type animals (Aman et al., 2019). The authors demonstrated that this effect was partially recovered by the opioid antagonist naloxone, being potentiated by morphine. It is quite interesting that transgenic mice with Alzheimer’s disease exhibited an elevation of plasma β-endorphin levels, under the induction of osteoarthritis. It was postulated that impaired activation of the opioid system underlies the changes of inflammatory pain in Alzheimer’s disease (Aman et al., 2019). Pharmacological strategies focused on these opioidergic pathways might be of relevance in the clinical setting. It can be also inferred that dysregulation of opioid transmission in dementia and chronic pain is implicated in additional comorbidities in the elderly, such as mood disorders.

### Depression

According to Zis et al. (2017), chronic pain and depression are prevalent in elderly and they have a bidirectional relationship. The authors concluded that depression and pain might be risk factors for each other and that robust epidemiologic studies about chronic pain and comorbid depression are lacking. Mossey and Gallagher (2004) showed that 13% of the elderly suffer from both depression and chronic pain. A strong association between pain severity and depression has been demonstrated in elderly (Roy and Thomas, 1986; Turk et al., 1995). Furthermore, the most important predictor of a higher pain levels in primary care old patients was chronic low back problems, especially if combined with chronic gastritis, hyperuricemia/gout, cardiac insufficiency, neuropathies, and depression (Scherer et al., 2016). A study examined whether depression mediates the relationship between pain and sleep disturbances in middle-aged and older adults. The authors concluded that depression partially mediated the relationship between day-to-day pain inconsistency and sleep efficiency, and total wake time, according to the evaluation of 82 community-dwelling older adults (Ravyts et al., 2019).

### Burning Mouth Syndrome

Some pain syndromes particularly affect elders. Such as, burning mouth syndrome (BMS) represents a chronic oral pain condition mainly affecting postmenopausal women, which has been correlated with xerostomia and reduced salivary rates, in addition to other complications, in spite of the absence of any mucosal lesion (Werfalli et al., 2021). Recent evidence has suggested that neuroinflammation is implicated in the pathophysiology of BMS, as patients exhibit increased plasma levels of several cytokines and chemokines, and the benefits of antidepressants in BMS-related pain are partly related to their ability to modulate the levels of inflammatory mediators (Miyauchi et al., 2019). Furthermore, BMS in the elderly has been associated with both cold and warm hypoesthesia, consistent with small fiber neuropathy, allied with genic alteration of dopamine D2 receptor (Kolkka et al., 2019). A retrospective population-based cohort study, including 586 patients with BMS diagnosis and 1,172 controls, demonstrated an increased incidence of anxiety and depression in BMS individuals, without any significant association with the risk of getting dementia or Parkinson’s disease (Kim J. Y. et al., 2020). Likewise, a study involving 52 women with BMS diagnosis, with a mean age of 63 years, revealed that pain intensity and pain interference positively correlated with anxiety and depression scores, besides the levels of preoccupation with the symptoms (Forssell et al., 2020; see Figure 2). Nonetheless, these pieces of evidence reinforce the notion that chronic pain states worsen neuropsychological aspects in seniors, requiring a multidisciplinary therapeutic approach for pain and psychological management. An important problem for the elders presenting with BMS is the complexity for diagnosis. The absence of any visible lesion has been associated with delayed or equivocal diagnosis, besides inappropriate pharmacological treatments. In this regard, a study conducted with 102 BMS women, with a mean age of 60 years, revealed that about 25% of individuals were misdiagnosed for candidiasis, and the mean time for diagnosis was 12 months, involving multiple health professionals (Freilich et al., 2020). This discussion is very important as many professionals do not take the elder’s complaints seriously, leading to mismanagement of painful symptoms, with the consequent aggravation of the psychological components in this population. Despite the absence of oral lesions associated with the burning sensation, a proteomic investigation of whole saliva demonstrated the presence of 100 proteins specifically in BMS patients, besides an upregulation of 158 other proteins under BMS diagnosis, with a relevance for altered neurotrophin pathways in the affected individuals (Krief et al., 2019). Further studies on the mechanisms underlying BMS will bring real advances for the treatment of BMS-related symptoms in the elderly. The most important message in this case is that pain in the elderly must be managed with extra awareness, preventing neglecting attitudes from health professionals, caregivers and family.

### Viral Infections

Pain has been described as one of the neurological manifestations of several viral infections. For instance, chikungunya virus infection has been associated with a series of long-term post-infection complications, such as chronic joint pain, with a relevance in older people (Calvo et al., 2021). Recently, the WHO classification of the clinical forms of chikungunya virus infection in the elderly has been updated, considering the relevance of the disease in the older individuals, as well the disease burden in endemic regions (Godaert et al., 2021). Notably, elderly is a well-recognized risk factor for varicella-zoster virus reactivation, contributing for post-herpetic neuropathy (PHN) in this population. The efforts should be focused on vaccination campaigns enrolling old individuals to prevent further post-herpetic complications. Nonetheless, only a small part of the old population has access to the schemes of herpes zoster vaccination. However, considering the long-lasting manifestations of PHN and the limited efficacy of the available pharmacological strategies, prevention strategies are likely the best strategy to be adopted (John and Canaday, 2017; Gruver and Guthmiller, 2022). Extending the relevance of recognizing viral infections as a possible source of pain in elderly, a case report described the occurrence of abdominal pain combined with prostration appetite loss, vomiting and diarrhea in a 77-year-old woman with a diagnosis of herpes simplex virus esophagitis (Custodio et al., 2021).

At this moment, it is imperative to consider COVID-19 pandemic as a relevant factor for painful complications in elders. An interesting case report described the occurrence of trigeminal neuralgia affecting the ophthalmic nerve division, in a 65-year-old man presenting with respiratory symptoms of SARS-CoV-2 infection, which has been solved after patient recovery (Molina-Gil et al., 2021). The most relevant question in this respect is whether elders that had been infected with the virus might present persistent painful alterations following recuperation. In this regard, a 70-year-old patient with previous diagnosis of type 2 diabetes and myasthenia gravis, that had critical SARS-CoV-2 infection, developed lumbar herpes zoster and post-herpetic neuralgia, with the persistence of intermittent painful symptoms even within 4 months after the lesion resolution and the continuous pharmacological treatment of pain (Cao et al., 2020). From this data, it appears that pre-existing comorbidities can aggravate painful symptom secondary to COVID-19 disease in elders. Additional studies are necessary to assess chronic pain levels after COVID-19 infection in seniors (see Figure 2).

## Pain Management in the Elderly

From the abovementioned evidence, it is obvious that chronic pain states greatly influence the life quality of elder individuals, even by preceding or exacerbating other comorbidities. Therefore, there is a current interest to identify new approaches for pain management in this population.

The pharmacological treatment of chronic pain is quite limited and often ineffective, beyond the adverse side effects, like those presented by the analgesic drugs. Elderly people with chronic pain are difficult to treat, as well chronic pain itself is a complex condition to manage. In addition, old people usually have comorbidities or associated diseases, and for this reason, they use different drugs simultaneously, generating complex drug interactions. The antidepressants recommended for chronic pain treatment include tricyclic antidepressants (TCAs) and serotonin and norepinephrine reuptake inhibitors (SNRIs) (Saarto and Wiffen, 2010). These drugs inhibit the reuptake of serotonin and norepinephrine, elevating the neurotransmitter contents in the synaptic cleft. The selective serotonin reuptake inhibitors (SSRIs) are less effective for pain relief, when compared with SNRIs (Sindrup and Jensen, 1999). SSRIs and SNRIs have been associated to a significant increase in fall events in the elderly (Coupland et al., 2011). TCAs need to be used cautiously in older adults, because they might induce the syndrome of inappropriate antidiuretic hormone secretion (SIADH) or hyponatremia (By the American Geriatrics Society Beers Criteria Update Expert Panel, 2019).

The anticonvulsants, such as carbamazepine, phenytoin, and valproic acid are used to treat neuropathic pain. Carbamazepine and oxcarbazepine are first choice drugs for pain relief in trigeminal neuralgia (Di Stefano et al., 2018). These drugs block sodium channels, abolishing nervous hyperexcitability, by modifying the membrane excitability (Davies, 1995). Gabapentinoids act on primary afferent excitability and block α2δ subunits of calcium channels (Tremont-Lukats et al., 2000). The latter group has been widely used in the treatment of neuropathic pain, as they have fewer side effects when compared to older anticonvulsants (Ross, 2000). Regarding the elderly treatment, the first-generation drugs increase the risk of hyponatremia and SIADH in this population (By the American Geriatrics Society Beers Criteria Update Expert Panel, 2019). As for gabapentinoids, even when considering the milder side effects, like dizziness, drowsiness, fatigue, and weight changes, they should be used with vigilance in elder individuals, beginning the treatment with low doses. It is important to highlight that gabapentinoids can have additive effects on respiratory depression induced by CNS depressant drugs, such as opioids, and even lead to death (By the American Geriatrics Society Beers Criteria Update Expert Panel, 2019). This combination treatment is often used in elderly people with comorbidities, requiring wariness in this population (Throckmorton et al., 2018).

The primary mechanism of action of non-steroidal anti-inflammatory drugs (NSAIDs) rely on the inhibition of prostaglandin synthesis, by preventing COX-1 and/or COX-2 activation and the arachidonic acid metabolism (Vane and Botting, 1998). Older NSAIDs, such as aspirin, ibuprofen, and naproxen are non-selective inhibitors of both COX-1 and COX-2 isoforms, whilst coxibs, namely celecoxib and parecoxib, selectively block COX-2 activation. NSAIDs are analgesic, anti-inflammatory, and antipyretic drugs, and they are effective in the treatment of painful diseases, with mild to moderate symptoms, which have an inflammatory component involved, such as musculoskeletal and osteoarthritis-related pain. However, they can lead to renal and gastrointestinal toxicity, causing nausea, diarrhea, mucosal damage, gastric ulcer, and dyspepsia (Shimp, 1998; Hawkey, 2000; Coxib and traditional Nsaid Trialists’ (CNT) Collaboration, Bhala et al., 2013). For this reason, the concomitant use of gastroprotective therapy is required (Yeomans et al., 1998). NSAIDs should be used with caution in the elderly, as they might have negative effects on cardiac comorbidities in this population, mainly concerning hypertension. In addition, NSAIDs can abolish the cardioprotective effects of aspirin, which is widely used by the elderly (Burnakis, 2002; Etminan and Samii, 2003; Schmidt et al., 2016). The selective COX-2 inhibitors, such as celecoxib, are expected to have fewer gastrointestinal side effects, while the long-lasting use has been associated with an increased risk of thromboembolic events, requiring further awareness in older individuals (Heim and Broich, 2006). Besides celecoxib, the continuous use of non-selective COX inhibitors, including diclofenac, ibuprofen, and meloxicam, have also been correlated with a higher risk of venous thromboembolism, in old adults presenting with knee osteoarthritis, an effect that was not observed for naproxen (Lee et al., 2016). In relation to post-operative persistent pain, it was observed that elder patients subjected to hepatectomy under general-epidural anesthesia, presented a significant reduction of pain average after treatment with the COX-2 inhibitor parecoxib, as evaluated by the visual analogic pain scale and the needing of rescue analgesic medication (Ge et al., 2021), indicating that this drug might be useful for pain control after extensive surgical procedures in senior individuals.

The opioid analgesic effects are attributed to their action on opioid receptors, mainly the μ and κ subtypes, expressed in PAG, spinal cord, joint synovium, and intestinal mucosa (Stein, 2016). Opioids have long-lasting analgesic effects in the elderly, when compared with young individuals (Bellville et al., 1971; Kaiko et al., 1982). However, they have side effects such as drowsiness and dizziness, which can lead to an increase in the number of falls and fractures in the elderly (Vestergaard et al., 2006). Oxycodone, in its oral formulation, at small doses, has been used for patients with swallowing problems, such as this is the case of elderly individuals (Schwan et al., 2019). One study showed that tramadol pharmacokinetics was little affected by age, as well as liver and renal function (Likar et al., 2006). However, opioids have been associated with hyponatremia or SIADH, and their judicious use is strongly recommended (By the American Geriatrics Society Beers Criteria Update Expert Panel, 2019). One of the opioids that have been highlighted for use in the elderly is transdermal buprenorphine, a partial μ agonist, and a κ and δ antagonist (Vadivelu and Hines, 2008). This is due to the less pronounced side effects when compared with other opioids, being safe for elderly patients with renal insufficiency (Griessinger et al., 2005; Muriel et al., 2005; Filitz et al., 2006). The potential for opioid dependence should be considered in the elderly population, as 70% of the elderly in geriatric homes receive opioids as a treatment for chronic non-cancer pain. Moreover, it is considered that 1–3% of older adults use opioids inappropriately (Campbell et al., 2010; Papaleontiou et al., 2010; Lapane et al., 2013), claiming attention for the opioid-related risks in elders, especially in nursing homes. In fact, recent data suggests high levels of opioid prescription for patients with dementia, related or not to Alzheimer’s disease, and the potential adverse pharmacological interactions with other central-acting drugs in this group (Wei et al., 2021).

Interventional local techniques like epidural steroid injections, lumbar facet injections, percutaneous vertebral augmentation, sacroiliac joint injections, and hip and knee joint injections are used as part of a multidisciplinary strategy, to decrease the need for systemic pharmacological therapy (Brooks and Udoji, 2016).

Taking into account the abovementioned evidence, it is feasible to conclude that pharmacological options currently available for treating chronic pain present a series of limitations when considering the elderly population, mainly regarding the drug interactions and the serious side effects. Therefore, there is an urgent need for new pharmacological and non-pharmacological approaches focused in the senior population. In this regard, a multidisciplinary approach to pain management is required to provide pain relief in older persons (Schwan et al., 2019). Hence, in addition to pharmacological treatments, non-pharmacological approaches have been demonstrated to be useful for management of chronic pain in elder patients. New therapies target the biopsychosocial aspects of pain, such as cognitive behavioral therapy, progressive relaxation, mindfulness meditation, and pain neuroscience education (PNE) (Morone and Greco, 2007; Beissner et al., 2009; Eccleston et al., 2016). PNE has provided interesting results in the chronic pain management in the elderly, according to Rufa et al. (2019). This intervention aims to educate patients about the complex factors that induce pain, thus changing the way that these individuals understand and face the painful condition (Moseley, 2002; Louw et al., 2016). The researchers demonstrated that PNE improved gait speed, pain disability, and fear of movement, in older adults with chronic back and/or lower extremity pain (Rufa et al., 2019).

Noteworthy, Kim K. S. et al. (2020) provided preliminary evidence indicating that art making can attenuate pain experience in > 50-year-old subjects, probably by favoring a positive mood. This is a reliable proposition considering the multidimensional levels of pain dynamics and the benefits of holistic therapies in this context, as proposed by the authors themselves. The psychological intervention of a 1-day Acceptance and Commitment Therapy (ACT) workshop was effective against persistent post-surgical pain and dysfunction in older veterans. The patients received treatment as Usual (TAU) or TAU plus a 1-day ACT workshop to prevent chronic pain and opioid use following orthopedic surgery. The ACT group reached pain alleviation (at 3 months post-orthopedic surgery) and opioid cessation (9 days earlier) sooner, when compared with the standard care group (Dindo et al., 2018).

Aging promotes musculoskeletal dysfunction due to the process of sarcopenia, with loss of muscular mass and strength (Janssen et al., 2002). Multiple modalities of rehabilitation/physical therapy have been shown to improve overall mobility and physical function in older people (Fiatarone et al., 1990; Gill et al., 2002; Frontera et al., 2003; de Vries et al., 2012). Elderly patients with hip or knee osteoarthritis showed an improvement of pain outcomes with resistance-based strengthening therapies (Kovar et al., 1992; Ettinger et al., 1997; van Baar et al., 1998). Regimens focused on high-intensity strengthening and low-intensity strengthening are effective to stimulate endurance increase in older individuals (Vincent et al., 2002). Concerning alternative therapies, a randomized controlled pilot study showed that the Tai Chi modality offered twice weekly for 12 weeks, had modest results in older adults with chronic multisite pain, although it significantly reduced the pain severity (You et al., 2018). Additionally, older adults who completed 70% or more classes of Tai Chi had a reduction of β-endorphin blood levels (You et al., 2020). Also, elderly people at risk for ischemic stroke reported improvement from neck pain, muscle pain, and low back pain after Tai Chi training (Zheng et al., 2017).

In a different perspective, a systematic review conducted by Perna et al. (2020) indicated that dietary monitoring, and the supplementation with specific nutrients and antioxidant agents can reduce the needing of analgesics for managing musculoskeletal pain in the elderly. However, the interactions between drugs and nutrients and/or herbal supplements cannot be overlooked in this population. According to Du et al. (2019), the consumption of 40 g freeze-dried blueberry powder daily for 4 months reduced pain and stiffness, and improved gait performance in adults aging from 45 to 79 years, presenting with symptomatic knee osteoarthritis. These effects can be explained by the anti-inflammatory properties of dietary polyphenols (Du et al., 2019). On the other hand, vitamin D and marine omega-3 fatty acid supplementation did not have any effect in older adults with chronic knee pain, as demonstrated by a double-blind and placebo-controlled trial (MacFarlane et al., 2020).

Simon et al. (2015) tested transcutaneous electrical nerve stimulation (TENS) in older individuals to treat chronic low back pain. The authors reported that older adults and younger adults gained similar responses for TENS treatment. However, older adults required higher TENS amplitude across all the sessions to experience episodic axial pain relief in comparison with younger adults (Simon et al., 2015). Another research group suggested that chemical moist heat right after the modality was able to reduce pain in 52.3% in older individuals, when compared with 30.5% in diabetes subjects and 33.3% in younger individuals (Petrofsky et al., 2011).

## Concluding Remarks

Primary and secondary chronic pain diseases are overly complex by nature. This is strengthened while analyzing chronic pain in the elderly. If institutionalization is added to this intricate scenery, it is still clearer that complexity is the word summarizing the main data discussed in the present review. It has been demonstrated that aging leads to alterations of sensorial components of pain—this is evidenced mainly by changes in pain thresholds—depending on the nature of stimulation, mostly in females. Moreover, there is controversial data on whether getting older affects pain coping mechanisms. As well, it is not obvious if central networks related with affective pain processing are positively or negatively impacted during aging. Even more elements can be added to the present discussion, as it has been demonstrated that chronic pain might aggravate neurodegenerative diseases inherent for the elderly, such as all-causes or Alzheimer’s disease-related dementia or Parkinson’s disease. In this group of elders, pain can aggravate specific disease signs, such as memory loss in Alzheimer’s or motor symptoms in Parkinson’s disease. Painful symptoms might well worsen neuropsychological symptoms, including mood and sleep disorders. Hence, chronic pain affects both autonomy and independence in seniors, greatly compromising their overall life quality when comorbidities are present. As another point of debate, some painful conditions mainly affect old ages, such as BMS, and health professionals need to be aware of the recent advances related to the pain mechanisms in this specific case. The same observation might be extended for COVID-19 elder survivors, for whom chronic pain has been described as a possible post-infection complication, despite the scarce evidence on this issue. Opportunely, new treatment alternatives, especially non-pharmacological approaches, have been emerging for the control of painful symptoms in the elderly, and this must be accelerated in a near future. All in all, it can be concluded that a lot remain to be studied in the theme of the present review—definitely, we still need to learn more about the mechanisms and the management of chronic pain in the elderly.

## Author Contributions

AD organized the topics and references and drew the figures. Both authors drafted, revised, and edited the manuscript.

## Conflict of Interest

The authors declare that the research was conducted in the absence of any commercial or financial relationships that could be construed as a potential conflict of interest.

## Publisher’s Note

All claims expressed in this article are solely those of the authors and do not necessarily represent those of their affiliated organizations, or those of the publisher, the editors and the reviewers. Any product that may be evaluated in this article, or claim that may be made by its manufacturer, is not guaranteed or endorsed by the publisher.
